# Circulating MicroRNAs in Patients with Vulvar Squamous Cell Carcinoma and Its Precursors

**DOI:** 10.3390/ncrna11010013

**Published:** 2025-02-07

**Authors:** Julia Rymuza, Angelika Długosz, Kamil Zalewski, Artur Kowalik, Mateusz Bujko, Magdalena Kowalewska

**Affiliations:** 1 Department of Molecular and Translational Oncology, Maria Sklodowska-Curie National Research Institute of Oncology, 02-781 Warsaw, Poland; julia.rymuza@nio.gov.pl (J.R.); zalewski81@gmail.com (K.Z.); mateusz.bujko@nio.gov.pl (M.B.); 2 Department of Immunology, Biochemistry and Nutrition, Medical University of Warsaw, 02-091 Warsaw, Poland; angelika.dlugosz@gmail.com; 3 Department of Molecular Diagnostics, Holycross Cancer Centre, 25-734 Kielce, Poland; artur.kowalik@onkol.kielce.pl; 4 Department of Genetic Engineering, Holy Cross Cancer Centre, 25-734 Kielce, Poland; 5 Division of Medical Biology, IB, Jan Kochanowski University, 25-406 Kielce, Poland

**Keywords:** vulvar squamous cell carcinoma, vulvar intraepithelial neoplasia, vulvar high-grade squamous intraepithelial lesions, circulating microRNA, miR-145, miR-221, miR-222

## Abstract

**Objectives**: Vulvar squamous cell carcinoma (VSCC) is a rare gynecologic malignancy, with most cases arising from differentiated vulvar intraepithelial neoplasia (dVIN). Approximately one-third of VSCC cases originate from high-grade squamous intraepithelial lesions (HSILs), which are associated with persistent infection by varieties of high-risk human papillomavirus (hrHPV). This study aimed to quantify the circulating microRNAs (miRNAs) in the plasma of patients with premalignant conditions (dVIN and HSILs) and VSCC using TaqMan Low-Density Arrays. **Methods**: Plasma samples were collected from 40 patients, including those treated for HSILs, dVIN, and VSCC. Quantitative real-time PCR (qRT-PCR) identified the circulating miRNAs differentially expressed in the plasma of VSCC patients compared to patients with precancerous lesions. **Results**: A total of 31 differentially expressed miRNAs (DEMs) were found to be significantly upregulated in plasma from VSCC patients compared to precancerous cases. None of the analyzed miRNAs were able to distinguish VSCC cases based on hrHPV tumor status. **Conclusions**: This study provides strong evidence that a distinct set of miRNAs can differentiate between plasma samples from VSCC patients and those with precancerous lesions. Thus, these DEMs have potential diagnostic and prognostic value. “Predisposing” DEMs could be developed as biomarkers to aid in the assessment of vulvar lesions, helping to exclude or confirm progression toward cancer.

## 1. Introduction

Vulvar carcinoma, a rare malignancy, accounts for 3 to 5% of gynecologic cancers. Vulvar squamous cell carcinoma (VSCC) is the most common type, comprising approximately 90% of malignant tumors in this location. Most vulvar carcinomas arise from differentiated vulvar intraepithelial neoplasia (dVIN) and follow a trajectory that is independent of the human papillomavirus (HPV) pathway, with *TP53* mutations being a hallmark of this pathway. Approximately one-third of vulvar carcinomas arise from high-grade squamous intraepithelial lesions (HSILs). They are associated with both persistent infection and with high-risk HPV (hrHPV) types, primarily HPV 16 and 18. For the discrimination of HPV-dependent and -independent vulvar precancers, the WHO provides specific histopathologic criteria and recommends immunohistochemical (IHC) assessment via p16 and TP53 staining [1]. The WHO “desirable” diagnostic criteria encompass p16 block-type IHC for HPV-dependent precancers and p16 negativity along with abnormal TP53 IHC for HPV-dependent lesions. p16 IHC is a surrogate marker for hrHPV infection, showing good correlation with HPV testing [2]. The rearrest subtypes are HPV-negative and wild-type TP53 VSCCs, with the suspected precursor termed verruciform acanthotic VIN verruciform acanthotic VIN (vaVIN) or vulvar aberrant maturation (VAM) [1,2].

Mutations in TP53 result in aberrant protein expression patterns, as assessed indirectly by IHC, with the best-known overexpression of TP53. Six major patterns of TP53 staining in VSCC (and vulvar precancers) were reported to be consistent with *TP53* wild-type and *TP53* mutant statuses [3]. Most studies report that HPV negativity and the abnormal expression of TP53 are associated with worse outcomes in patients with VSCC [2]. The WHO recommends documenting the type of VSCC (HPV-associated or HPV-independent) on the pathology reports [1]. However, due to considerable histomorphological overlap between the two types, this distinction may be challenging [4]. Therefore, the International Collaboration on Cancer Reporting recommends using p16 IHC for an accurate classification of VSCC into the two main groups: HPV-associated and HPV-independent VSCC [4]. The International Federation of Gynecology and Obstetrics (FIGO) advocates that all vulvar cancers be tested for both HPV and p16 [5]. Moreover, an algorithm for the application of p16 and TP53 IHC in VSCC classification was proposed [2]. However, it was not a formal recommendation. Nevertheless, patients with these etiopathogenic VSCC types are currently treated in the same way. The presence of dermatoses, such as lichen sclerosus or lichen planus, low-grade SIL (LSIL), or coexistent precursor lesions, in the setting of vulval VSCC further complicates histopathological evaluation [6].

As with other rare cancers, our understanding of the biology of VSCC remains limited [7]. In this context, in the past decade, research has been undertaken on the role of microRNAs (miRNAs) in the biology of vulvar carcinoma. The initial study by de Melo Maia et al. [8] identified miRNAs with altered expression patterns in VSCC tumors. Some of these alterations were correlated with clinicopathological characteristics in patients with VSCC, such as lymph node metastases (the downregulation of miR-223-5p and miR-19-b1-5p), vascular invasion (the downregulation of miR-100-3p and miR-19-b1-5p), HPV infection (the upregulation of miR-1274b and downregulation of miR-519b), or advanced disease stage (miR-519b and miR-133a overexpression). The same group [9] demonstrated the association of miR-20a and miR-106a upregulation with deeper tumor invasion in VSCC. The upregulation of another miR, miR-590-5p, was documented as being associated with lymphatic metastases [8]. Recently, a predisposing miRNA profile consisting of 21 miRNAs was established in order to predict the risk of developing VSCC in a background of vulvar lichen sclerosus, a chronic inflammatory disorder of unknown etiology [10]. These studies confirm that the dysregulation of miRNA expression in tumors is implicated in vulvar carcinogenesis and shed light on their potential functions as oncogenes or tumor suppressors in VSCC. 

miRNAs hold promise as tumor biomarkers that are detectable in blood via a minimally invasive liquid biopsy approach. miRNAs are the most examined ncRNAs in human plasma due to their relative stability and standardized detection methods [11,12]. Circulating miRNAs, present in human plasma or serum, can exist in several forms—free circulating miRNA and those encapsulated within extracellular vesicles or bound to proteins. Each form contributes to stability and biological activity. To our knowledge, our study on miR-431-5p is the first study to report on miRNA levels in the blood of patients with VSCC. We found that miR-431-5p levels were increased in plasma from patients with VSCC compared to those with vulvar precancers. Low levels of circulating miR-431-5p were found to be indicative of unfavorable survival in patients with VSCC [13]. In this study, we aimed to determine the circulating microRNA profile in plasma samples of VSCC patients using TaqMan Low-Density Arrays and to compare the results with circulating microRNAs in patients with vulvar precancer lesions.

## 2. Results

Based on the Ct results obtained for the three endogenous controls—U6 snRNA, RNU44, RNU48—included in the TaqMan MicroRNA Arrays, U6 snRNA was selected for qPCR data normalization. RNU44 was not found to be present in all the analyzed samples, while the detection of RNU48 was inconsistent across all the cards and samples, and thus these two molecules were excluded from further analysis. The results were normalized with U6 snRNA expression. MiRs that were absent from all the samples analyzed were excluded, leaving 116 miRNAs for further assessment. Samples without U6 snRNA expression were excluded from analysis. Finally, the discrimination abilities of the 116 miRNAs that underwent filtering were assessed for their discriminative abilities between 10 precancer samples (5 HSILs and 5 dVIN) and 27 VSCC samples (12 hrHPV-negative, 14 hrHPV-positive and one of unknown hrHPV status). The basic demographic data of these patients are provided in Table 1, whereas the qPCR data are stored in the Zenodo repository at https://zenodo.org/records/14672217 (accessed on 1 February 2025).

**Figure 1:**
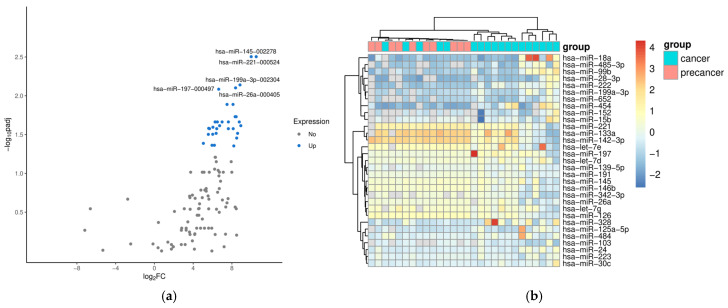
A volcano plot (**a**) and heat map (**b**) for differentially expressed miRNAs between precancer and VSCC groups. (**a**) The significant DEMs are highlighted with blue dots, and the top five upregulated are marked with miR symbols. (**b**) A heat map of significant differentially expressed miRNAs. The heat map shows the signal of 31 miRNAs (*p*-value < 0.05).

Data comparison between precancer and VSCC groups revealed 31 differentially expressed miRNAs (DEMs) with statistical significance. A volcano graph for these DEMs is presented in Figure 1a. This shows the distribution of significance [–log10(*p*-value)] vs. fold change [log2(fold change)], demonstrating that 31 miRNAs were significantly differentially expressed between the precancer group and the VSCC group (*p* < 0.05). The heat map and hierarchical clustering of significant DEMs are shown in Figure 1b. Normalized levels of miRNA were used to hierarchically cluster samples. We observed samples from patients with precancers (dVIN or HSILs) to be clustered closer together, and the VSCC group was separated quite well from the precancer group. None of the analyzed miRNAs were found to differentiate samples from patients with VSCC based on tumor hrHPV status. Additionally, we performed miRNA target prediction with the TargetScan database v. 8.0 and overrepresentation analysis to assess the gene enrichment in functional categories. The Gene Ontology (GO) and the Kyoto Encyclopedia of Genes and Genomes (KEGG) terms identified for at least 10 out of 31 DEMs were considered to be common processes and are listed in Table S1. The result of the cluster analysis of these terms is presented in Figure S1.

**Figure 2:**
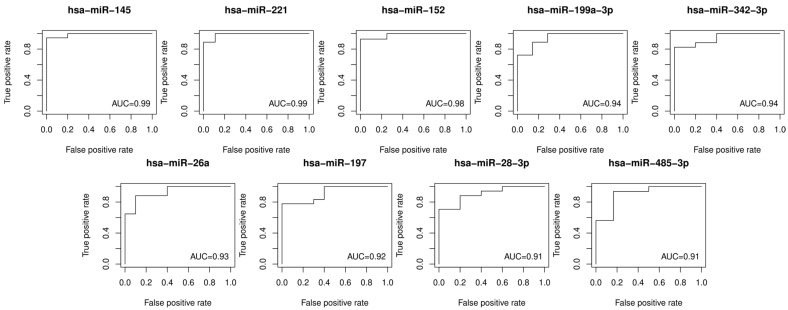
Receiver operating characteristic curves for the discrimination of vulvar precancers and cancers based on the plasma levels of the top DEMs (AUC > 0.9). AUC: area under the ROC curve; ROC: receiver operating characteristic.

The ROC (receiver operating characteristic) curves for 31 DEMs were generated in order to examine their diagnostic accuracies. The log-rank and ROC AUC tests were performed to assess differences in DEM levels in the two sample groups (plasmas obtained from patients with vulvar precancer vs. cancer) and the discriminating ability of each miRNA. The ROC curves for DEMs with AUC values higher than 0.9 are shown in Figure 2. The logarithm of fold changes, adjusted *p*-values for the Mann–Whiteny test, and AUC values from the ROC tests for DEMs are presented in Table 2.

**Figure 3:**
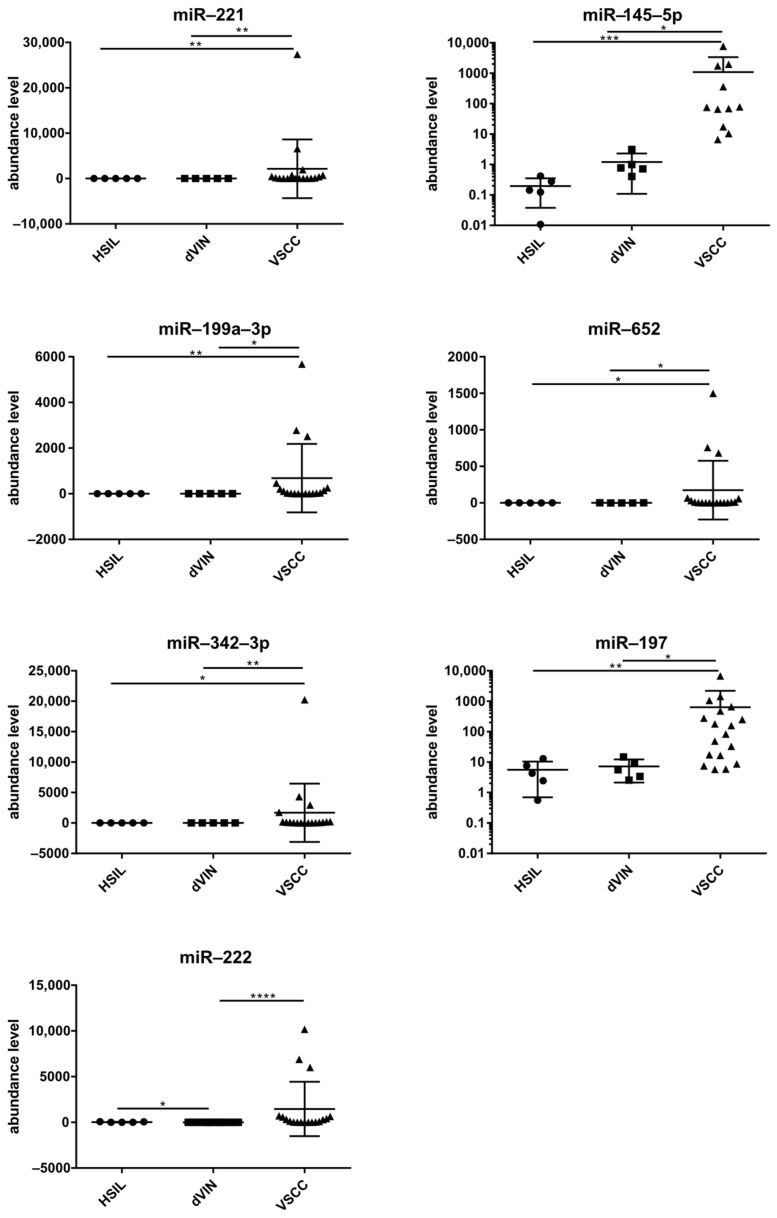
Levels of seven selected miRNAs (mean ± SD) in plasma samples of patients with vulvar precancers and cancers. Significant alternations are indicated by asterisks (* *p*-value ≤ 0.05; ** *p*-value ≤ 0.01; *** *p*-value ≤ 0.001; **** *p*-value ≤ 0.0001).

The significance of the differences in levels of DEMs between the three sample groups, i.e., plasma samples from patients with HSILs, dVIN, and VSCC, was examined with the Kruskal–Wallis test and post hoc Dunn’s multiple comparison analysis. Figure 3 depicts the results for seven miRs with the best discriminatory performances for HSILs, dVIN, and VSCC samples. Of note, in this small sample group, miR-222 levels in plasma samples differed between patients with HSILs and dVIN. 

## 3. Discussion

MiRNAs are small noncoding RNA molecules that influence various cellular processes by modulating the expression of target genes. In cancer, miRNAs can function as oncogenes (oncomiRs) or tumor suppressors, and their aberrant expression profiles are associated with various types of tumors, including female genital tract tumors [14]. In vulvar carcinoma, specific miRNAs are associated with tumorigenesis. Some preliminary results of in vitro experiments suggested the use of an miRNA-based therapeutic option against VSCC [9]. However, these preliminary data require further verification and more information on the role of specific miRs in vulvar carcinogenesis. For example, the downregulation of miR-223-5p was found to be correlated with lymph node metastases in VSCC patients [8], while another study [15] described miR-223-5p as an oncomiR in vulvar carcinoma providing a putative mechanism of its function in tumor invasion.

Currently, information regarding circulating miRNAs in VSCC is limited. Yet, these molecules hold potential as diagnostic biomarkers, particularly in the context of liquid biopsy, where their relative stability in biological fluids allows for the non-invasive detection and monitoring of tumor-specific molecular changes. Previously, our group demonstrated that lowered levels of circulating miR-431-5p are associated with poor prognosis in patients with VSCC, supporting the potential utility examination of miRs in the blood in order to identify patients at high risk of progression [13]. By analyzing miRNA abundance patterns in the blood of patients treated for HSILs, dVIN, and VSCC, this study aimed to identify circulating miRNAs with qRT-PCR to aid in distinguishing between malignant and premalignant conditions via plasma analysis. Our analysis demonstrated the differential levels of circulating miRNAs between patients with vulvar premalignant lesions and carcinoma. Our study revealed that among the analyzed miR cases, hsa-miR-221-3p and hsa-miR-145-5p were highly upregulated in plasmas of the VSCC group compared to the premalignant group. We also provided the results of in silico miRNA target prediction and biological pathway analyses. However, as the source of identified circulating miRs in plasma samples of the patients enrolled in our study remains unknown, these data should be interpreted with caution.

Chief among upregulated DEMs in a set of genital tumors, miR-221 is an extensively investigated oncomiR that plays an important role in the progression of many malignancies [16]. Of interest, apart from targeting mRNAs involved in main survival pathways, it can induce macrophage tolerization by silencing inflammatory genes [17]. miR-221 was shown to target *TP53BP2*, a positive regulator of the TP53 pathway [14]. miR-221 has potential as a diagnostic and prognostic biomarker, with its detection in body fluids offering a promising liquid biopsy tool. It is also a therapeutic target of various reported inhibition strategies, including the locked nucleic acid (LNA) inhibitor examined in an in-human study [13]. miR-221 is encoded by one miR-221/222 gene cluster with miR-222, another DEM identified in our study. Interestingly, miR-222 levels in plasma samples were revealed to differentiate patients with HSILs and dVIN. This finding promising in the context of diagnostic value of miR-222, although statistically significant, should be interpreted with caution due to the small sample numbers examined.

On the contrary, the second of the top upregulated DEMs in the blood of VSCC patients, miR-145, is a well-established tumor suppressor [18]. A wide range of cancers are characterized by reduced levels of this microRNA. It was demonstrated that miR-145-5p can regulate cell proliferation and migration by modulating various signaling pathways, such as MAPK and PI3K/AKT [19]. Due to the anti-oncogenic effects of miR-145, the therapeutic approaches proposed in oncology involve restoring its expression or using its mimics. Given the identified role of miR-145 as a tumor suppressor, our finding of its upregulations seems unexpected. Furthermore, miR-145 was shown to suppress ovarian cancer cell invasion and migration by targeting i.a. *HMGA2* (High-Mobility Group AT-Hook 2) gene [20]. Our group has previously documented an increase in HMGA2 protein abundance in VSCC tumors as compared to vulvar premalignant lesions [21]. However, in the blood of cancer patients, miR-145 levels can be significantly reduced, as recently reported for head and neck squamous cell carcinoma [22]. The mechanistic biological explanation for the large increase in miR-145 in the blood of VSCC patients remains to be solved. The same notion is true for all identified DEMs that can be used to discriminate patients with VSCC from patients with precursor lesions. Our data provide preliminary information on the detection of miRNAs that are potentially involved in early vulvar carcinogenesis processes. 

The role of miRNAs in the progression from premalignant conditions to cancer underscores their potential as prognostic ‘early warning’ biomarkers, as recently demonstrated in cervical malignancies through the examination of miRNA abundance and the methylation status of their genes in cytologic or tissue samples [23,24]. Many microRNA molecules—including miR-221, miR-222, and miR-145, as mentioned above—are present in the blood of cancer-free individuals and can be dysregulated in those with other medical conditions. Therefore, prior to developing new tests, case–control studies including numerous control samples are needed to assess the reliability of candidate new biomarkers. For instance, increased miR-145 plasma levels were recently reported to be associated with a decreased risk of venous thromboembolism in this type of a study [25]. Such studies are necessary when searching for new biomarkers for vulvar cancer patients, which is challenging due to the rarity of this disease.

## 4. Materials and Methods

### 4.1. Study Populations

Serum samples were obtained from 40 patients treated for premalignant vulvar lesions (HSILs, *n* = 7 and dVIN, *n* = 6) and VSCC (*n* = 27) at the Maria Sklodowska-Curie National Research Institute of Oncology Warsaw, Poland, and at the Holycross Cancer Center in Kielce, Poland, between December 2004 and March 2017. The cancer patients enrolled in the study had microscopically confirmed primary VSCC in the early stages (FIGO stage I, *n* = 18) and advanced stages (FIGO stage III, *n* = 9), and were aged 46 to 83 (median age–68). The basic demographic data of the patients included in the final analysis are provided in Table 1.

### 4.2. HPV Genotyping

The HPV status of precancer lesions and vulvar malignant tumors was determined, as described previously [26], using the AmpliSens HPV HCR-genotype-titre-FRT test. This detects 14 hrHPV genotypes, namely, HPV16, 18, 31, 33, 35, 39, 45, 51, 52, 56, 58, 59, 66, and 68, following the manufacturer’s protocol (InterLabService Ltd. (Moscow, Russia), Cat. No. R-V67-F-CE). 

### 4.3. RNA Isolation from Plasma and qPCR

Plasma samples obtained from patients and healthy donors were banked at −70 °C before their analysis. Total RNA was isolated from 200 µL of plasma using the miRNeasy Serum/Plasma Kit (Qiagen (Hilden, Germany), Cat. No. 217184). Then, 100 ng of total RNA was used for miRNA reverse transcription, using Pool A of Megaplex™ RT Human Primers (Applied Biosystems (Waltham, MA, USA), Cat. No. 4373360) in the Veriti™ 96 Well Thermal Cycler (Applied Biosystems). The miRNA cDNA was then preamplified using Megaplex™ PreAmp Primers, Pool A, in the Gene Amp^®^ PCR System 9700 (Applied Biosystems, Cat. No. 4391128). Following dilution, preamplified microRNA cDNA was loaded onto TaqMan^®^ MicroRNA Arrays A v2.0 (Applied Biosystems, Cat. No. 4444971) containing 384 TaqMan^®^ MicroRNA Assays per card. The qRT-PCR amplification and data acquisition were performed on the ViiA™ 7 Real-Time PCR System (Thermo Fisher Scientific|Applied Biosystems, Waltham, MA, USA). These microfluidic cards allow the quantification of 377 human microRNAs. The reverse transcription, pre-amplification, dilution, and qPCR steps were performed according to the manufacturer’s protocols (Applied Biosystems).

### 4.4. Data Analysis

The collected data were analyzed using threshold-cycle (Ct) values for the miRNAs with the Relative Quantification (RQ) Application Module on the Thermo Fisher Cloud. This was performed using automated baseline and manually set threshold values. The results were normalized, with endogenous controls included in the TaqMan MicroRNA Arrays. Ct values were exported and the relative amounts of each miRNA in the plasma of patients were calculated using the 2^−ΔCtT^ method. 

### 4.5. Statistical Analyses

The Mann–Whitney test was applied to find differentially expressed miRNAs between two sample groups. As a marker, miRNA performance was assessed using the area under the curve of the receiver operating characteristic curve (ROC AUC). Pearson correlation was calculated to check the association between miRNA expression and HPV status. These analyses were performed using python library scipy 1.13 [27]. Potential target genes for each miRNA were identified using the TargetScan database [28]. Over-representation analyses were conducted, using r library clusterProfiler [29], for the target gene lists of each miRNA, utilizing the Gene Ontology (GO) database [30] and the Kyoto Encyclopedia of Genes and Genomes (KEGG) database [31].

The nonparametric Kruskal–Wallis test and the post hoc Dunn’s multiple comparison test were used to assess the significance of differences in levels of selected miRs between the three sample groups. This statistical analysis was performed and the results were visualized using GraphPad Prism 6.07 (GraphPad Software, Boston, MA, USA). Differences at *p* < 0.05 were considered significant.

## 5. Conclusions

In summary, despite a small sample size, our study provides a strong indication that there is a set of miRNAs differentiating between plasma from patients with vulvar carcinoma and its precancer lesions. Distinguishing between VSCC and its precancers is critically important for patient management and outcomes. Early and accurate differentiation can prevent the overtreatment of benign or precancerous conditions and ensure the timely treatment of malignancies. In our study, several circulating miRNAs, particularly hsa-miR-221-3p and hsa-miR-145-5p, showed significantly increased levels in plasma of VSCC patients as compared to plasma samples of women with vulvar premalignant lesions. This finding suggests the potential diagnostic value of these molecules. Furthermore, understanding the molecular differences between these conditions, such as those reflected in circulating biomarkers—such as microRNAs—may improve diagnostic precision and guide the development of personalized therapeutic strategies. Further, the question of whether the profile of circulating miRs or specific miRs can be useful in predicting the progression of vulvar lesions to VSCC should be investigated.

## Figures and Tables

**Table 1:** Basic demographic data of the patients included in the analysis.

	Patients (*n*)	Median Age (Years) (Range)	FIGO	G	hrHPV Status
HSIL	5	64.1 (47.7–83.4)			N/A
dVIN	5	59.7 (41.1–79.5)			N/A
VSCC	27	68.6 (46.1–83.4)	I *n* = 18 III *n* = 10	G1 *n* = 10 G2 *n* = 12 G3 *n* = 5	negative *n* = 13 positive *n* = 14

Abbreviations: dVIN, differentiated-type vulvar intraepithelial neoplasia; FIGO, the International Federation of Gynecology and Obstetrics; G, tumor grade; hrHPV, high-risk human papilloma virus; HSILs, high-grade squamous intraepithelial lesions; N/A, not analyzed.

**Table 2:** Quantitative and statistical measurement of 31 high-confidence miRNAs.

miRNA	log2FC	padj	AUC
hsa-miR-145-5p	9.99	0.0031	0.99
hsa-miR-221-3p	10.51	0.0031	0.99
hsa-miR-152	8.44	0.0186	0.98
hsa-miR-199a-3p	8.83	0.0072	0.94
hsa-miR-342-3p	8.33	0.0186	0.94
hsa-miR-26a	8.38	0.0079	0.93
hsa-miR-197	6.64	0.0082	0.92
hsa-miR-485-3p	7.64	0.0217	0.91
hsa-miR-28-3p	8.14	0.0265	0.91
hsa-miR-15b	8.92	0.0243	0.90
hsa-miR-133a	8.11	0.0129	0.90
hsa-miR-191	7.50	0.0129	0.89
hsa-miR-652	8.59	0.0305	0.89
hsa-miR-103	8.49	0.0350	0.89
hsa-miR-139-5p	6.50	0.0217	0.87
hsa-let-7e	8.76	0.0217	0.86
hsa-miR-126	6.99	0.0217	0.86
hsa-miR-30c	6.26	0.0217	0.86
hsa-let-7d	6.73	0.0243	0.84
hsa-let-7g	5.60	0.0263	0.84
hsa-miR-484	6.04	0.0254	0.84
hsa-miR-222	6.37	0.0305	0.83
hsa-miR-99b	5.57	0.0314	0.83
hsa-miR-146b	7.63	0.0265	0.83
hsa-miR-223	6.43	0.0265	0.83
hsa-miR-328	5.69	0.0265	0.83
hsa-miR-24	6.02	0.0313	0.82
hsa-miR-454	5.05	0.0410	0.81
hsa-miR-125a-5p	6.19	0.0434	0.81
hsa-miR-18a	5.95	0.0434	0.80
hsa-miR-142-3p	8.20	0.0443	0.79

Abbreviations: log2 FC: Log2 fold-change; padj: adjusted *p*-value; AUC: area under the ROC curve.

## Data Availability

Data are available upon reasonable request from the corresponding author.

## Supplementary Materials

The following supporting information can be downloaded at: https://www.mdpi.com/article/10.3390/ncrna11010013/s1, Figure S1: KEGG and Gene Ontology terms for predicted target genes of differentially expressed miRNAs; Table S1: The top terms from over representation analysis from Gene Ontology (GO) and the Kyoto Encyclopedia of Genes and Genomes (KEGG) identified for differentially expressed miRNAs.

## References

[1] WHO Classification of Tumours Editorial Board (2020). Female genital tumours. WHO Classification of Tumours Series.

[2] Yang H., Almadani N., Thompson E.F., Tessier-Cloutier B., Chen J., Ho J., Senz J., McConechy M.K., Chow C., Ta M. (2023). Classification of Vulvar Squamous Cell Carcinoma and Precursor Lesions by p16 and p53 Immunohistochemistry: Considerations, Caveats, and an Algorithmic Approach. Mod. Pathol..

[3] Tessier-Cloutier B., Kortekaas K.E., Thompson E., Pors J., Chen J., Ho J., Prentice L.M., McConechy M.K., Chow K., Proctor L. (2020). Major p53 immunohistochemical patterns in in situ and invasive squamous cell carcinomas of the vulva and correlation with TP53 mutation status. Mod. Pathol..

[4] Rakislova N., Clavero O., Alemany L., Saco A., Quirós B., Lloveras B., Alejo M., Pawlita M., Quint W., Del Pino M. (2017). Histological characteristics of HPV-associated and -independent squamous cell carcinomas of the vulva: A study of 1,594 cases. Int. J. Cancer.

[5] Olawaiye A.B., Cotler J., Cuello M.A., Bhatla N., Okamoto A., Wilailak S., Purandare C.N., Lindeque G., Berek J.S., Kehoe S. (2021). FIGO staging for carcinoma of the vulva: 2021 revision. Int. J. Gynaecol. Obstet..

[6] McCluggage W.G., Bosse T., Focchi G. (2023). Carcinoma of the Vulva Histopathology Reporting Guide.

[7] Chehade R., Jerzak K.J., Tavanger F., Plotkin A., Gien L.T., Leung E., Mackay H. (2025). Advances in Vulvar Cancer Biology and Management. J. Clin. Oncol..

[8] de Melo Maia B., Lavorato-Rocha A.M., Rodrigues L.S., Coutinho-Camillo C.M., Baiocchi G., Stiepcich M.M., Puga R., de A.L.L., Soares F.A., Rocha R.M. (2013). microRNA portraits in human vulvar carcinoma. Cancer Prev. Res..

[9] de Melo Maia B., Ling H., Monroig P., Ciccone M., Soares F.A., Calin G.A., Rocha R.M. (2015). Design of a miRNA sponge for the miR-17 miRNA family as a therapeutic strategy against vulvar carcinoma. Mol. Cell. Probes.

[10] Borghi A., D’Abundo L., Bassi C., Lupini L., Tagliatti V., Zedde P., Lanza G., Gafa R., Negrini M., Corazza M. (2023). A microRNA signature to predict risk progression of vulvar lichen sclerosus to squamous cell carcinoma. Br. J. Dermatol..

[11] Pozniak T., Shcharbin D., Bryszewska M. (2022). Circulating microRNAs in Medicine. Int. J. Mol. Sci..

[12] Gayosso-Gómez L.V., Ortiz-Quintero B. (2021). Circulating MicroRNAs in Blood and Other Body Fluids as Biomarkers for Diagnosis, Prognosis, and Therapy Response in Lung Cancer. Diagnostics.

[13] Bujko M., Zalewski K., Szczyrek M., Kowalik A., Boresowicz J., Dlugosz A., Goryca K., Gozdz S., Kowalewska M. (2021). Circulating Hsa-miR-431-5p as Potential Biomarker for Squamous Cell Vulvar Carcinoma and Its Premalignant Lesions. Diagnostics.

[14] Ali A., Grillone K., Ascrizzi S., Carida G., Fiorillo L., Ciliberto D., Staropoli N., Tagliaferri P., Tassone P., Di Martino M.T. (2024). LNA-i-miR-221 activity in colorectal cancer: A reverse translational investigation. Mol. Ther. Nucleic Acids.

[15] Tassone P., Di Martino M.T., Arbitrio M., Fiorillo L., Staropoli N., Ciliberto D., Cordua A., Scionti F., Bertucci B., Salvino A. (2023). Safety and activity of the first-in-class locked nucleic acid (LNA) miR-221 selective inhibitor in refractory advanced cancer patients: A first-in-human, phase 1, open-label, dose-escalation study. J. Hematol. Oncol..

[16] Di Martino M.T., Arbitrio M., Caracciolo D., Cordua A., Cuomo O., Grillone K., Riillo C., Carida G., Scionti F., Labanca C. (2022). miR-221/222 as biomarkers and targets for therapeutic intervention on cancer and other diseases: A systematic review. Mol. Ther. Nucleic Acids.

[17] Seeley J.J., Baker R.G., Mohamed G., Bruns T., Hayden M.S., Deshmukh S.D., Freedberg D.E., Ghosh S. (2018). Induction of innate immune memory via microRNA targeting of chromatin remodelling factors. Nature.

[18] Xu W.X., Liu Z., Deng F., Wang D.D., Li X.W., Tian T., Zhang J., Tang J.H. (2019). MiR-145: A potential biomarker of cancer migration and invasion. Am. J. Transl. Res..

[19] Rahman M.S., Ghorai S., Panda K., Santiago M.J., Aggarwal S., Wang T., Rahman I., Chinnapaiyan S., Unwalla H.J. (2025). Dr. Jekyll or Mr. Hyde: The multifaceted roles of miR-145-5p in human health and disease. Non-Coding RNA Res..

[20] Kim T.H., Song J.Y., Park H., Jeong J.Y., Kwon A.Y., Heo J.H., Kang H., Kim G., An H.J. (2015). miR-145, targeting high-mobility group A2, is a powerful predictor of patient outcome in ovarian carcinoma. Cancer Lett..

[21] Fatalska A., Rusetska N., Bakula-Zalewska E., Kowalik A., Zieba S., Wroblewska A., Zalewski K., Goryca K., Domanski D., Kowalewska M. (2020). Inflammatory Proteins HMGA2 and PRTN3 as Drivers of Vulvar Squamous Cell Carcinoma Progression. Cancers.

[22] Yadav A.K., Singh N., Yadav S.K., Bhatt M.L.B., Pandey A., Yadav D.K., Yadav S. (2023). Expression of miR-145 and miR-18b in Peripheral Blood Samples of Head and Neck Cancer Patients. Indian. J. Clin. Biochem..

[23] Suvanasuthi R., Therasakvichya S., Kanchanapiboon P., Chamras Promptmas C., Chimnaronk S. (2025). Analysis of precancerous lesion-related microRNAs for early diagnosis of cervical cancer in the Thai population. Sci. Rep..

[24] Peronace C., Cione E., Marisol Abrego-Guandique D., De Fazio M., Panduri G., Caroleo M.C., Cannataro R., Minchella P. (2024). FAM19A4 and hsa-miR124-2 Double Methylation as Screening for ASC-H- and CIN1 HPV-Positive Women. Pathogens.

[25] Morelli V.N., Snir O., Hindberg K.D., Hveem K., Brækkan S.K., Hansen J.-B. (2024). High microRNA-145 plasma levels are associated with decreased risk of future incident venous thromboembolism: The HUNT study. Blood.

[26] Zieba S., Kowalik A., Zalewski K., Rusetska N., Goryca K., Piascik A., Misiek M., Bakula-Zalewska E., Kopczynski J., Kowalski K. (2018). Somatic mutation profiling of vulvar cancer: Exploring therapeutic targets. Gynecol. Oncol..

[27] Virtanen P., Gommers R., Oliphant T.E., Haberland M., Reddy T., Cournapeau D., Burovski E., Peterson P., Weckesser W., Bright J. (2020). SciPy 1.0: Fundamental algorithms for scientific computing in Python. Nat. Methods.

[28] McGeary S.E., Lin K.S., Shi C.Y., Pham T.M., Bisaria N., Kelley G.M., Bartel D.P. (2019). The biochemical basis of microRNA targeting efficacy. Science.

[29] Xu S., Hu E., Cai Y., Xie Z., Luo X., Zhan L., Tang W., Wang Q., Liu B., Wang R. (2024). Using clusterProfiler to characterize multiomics data. Nat. Protoc..

[30] Aleksander S.A., Balhoff J., Carbon S., Cherry J.M., Drabkin H.J., Ebert D., Feuermann M., Gaudet P., Harris N.L., The Gene Ontology Consortium (2023). The Gene Ontology knowledgebase in 2023. Genetics.

[31] Kanehisa M., Furumichi M., Sato Y., Matsuura Y., Ishiguro-Watanabe M. (2025). KEGG: Biological systems database as a model of the real world. Nucleic Acids Res..

