# Supplementary figures and images for: Circulating MicroRNAs in Patients with Vulvar Squamous Cell Carcinoma and Its Precursors
Source: Noncoding RNA. 2025 Feb 7;11(1):13. doi: 10.3390/ncrna11010013 (PMC11858568; doi:10.3390/ncrna11010013)

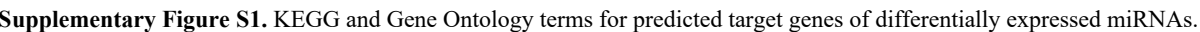

Supplement: Supplementary file 1 [file ncrna-11-00013-s001.zip › Supplementary Figure S1.pdf]
